# Psychological distress 12 years following injury in New Zealand: findings from the Prospective Outcomes of Injury Study-10 years on (POIS-10)

**DOI:** 10.1186/s40621-023-00419-8

**Published:** 2023-02-14

**Authors:** Helen E. Owen, Ari Samaranayaka, Emma H. Wyeth, Sarah Derrett

**Affiliations:** 1 Ngāi Tahu Māori Health Research Unit, Division of Health Sciences, University of Otago, PO Box 56, Dunedin, 9054 New Zealand; 2 Division of Health Sciences, Biostatistics Centre, University of Otago, PO Box 56, Dunedin, 9054 New Zealand

**Keywords:** Injury outcomes, Psychological distress, Kessler-6, Long-term outcomes, Predictors, Māori, New Zealand

## Abstract

**Background:**

Injuries can have detrimental impacts on mental health, even after physical recovery. In our Prospective Outcomes of Injury Study (POIS), 25% of participants experienced psychological distress (assessed using the Kessler 6) three months after a sentinel injury event (SIE), declining to 16% at 24 months post-SIE. Internationally, studies of hospitalised patients found distress persisted beyond 24 months post-injury and remained higher than the general population. However, most studies only assessed distress at one timepoint, relied on long-term recall, or were limited to small samples or specific injury types. Therefore, we aim to describe the prevalence of psychological distress 12 years post-SIE and to investigate pre-injury, injury-related and early post-injury characteristics associated with long-term distress.

**Methods:**

POIS is a longitudinal cohort study of 2856 New Zealanders injured between 2007 and 2009, who were on the national injury insurer, Accident Compensation Corporation entitlement claims’ register. Of these, 2068 POIS participants completed an interview at 24 months and agreed to further contact. They were invited to a follow-up interview 12 years post-SIE which included the Kessler-6 (K6), the psychological distress outcome of interest. Data about a range of pre-injury, injury-related and early (3 months) post-injury characteristics were collected via earlier interviews or administrative data sources (e.g. hospital discharge data).

**Results:**

Twelve years post-SIE, 1543 (75%) people were re-interviewed and 1526 completed the K6; *n* = 177 (12%) reported psychological distress. Multivariable modified Poisson regression models found pre-injury characteristics were associated with an increased risk of clinically relevant distress at 12 years, i.e. having inadequate income, identifying as Māori, Pacific or Asian and having one mental health condition. Early post-injury psychological distress and dissatisfaction with social relationships also increased risk. However, being older was associated with a reduced risk of distress.

**Conclusion:**

Clinically relevant distress persists long-term post-injury among adults with varying injury severity, types and causes, and at higher prevalence than in the general population. Early identification of injured people at risk of long-term psychological distress provides opportunities for timely interventions to reduce psychological distress.

## Background

Injury is a leading cause of disability globally (Rehm and Shield 2019), and injured people are at risk of poorer mental health, including psychological distress immediately after, as well as in the months and even years following the injury event (Saunders et al. 2012; Wiseman et al. 2015). Psychological distress refers to emotional suffering, and is typically characterised by symptoms of anxiety, depression and post-traumatic stress (Kendrick et al. 2017; Pratt 2009). Early post-injury symptoms of distress are known to be associated with poorer health-related quality of life and physical functioning at 12 months post-injury (Kendrick et al. 2017; O'Donnell et al. 2008), and with ongoing injury-related problems at 24 months post-injury (Wilson et al. 2017). People who experience early post-injury distress are also less likely to return to pre-injury levels in terms of work or health status compared to those not experiencing early distress (Richmond et al. 2009). The impacts of injury on psychological distress up to 12 months post-injury have been well documented and include an increased severity of distress symptoms (Wiseman et al. 2015) and occurrence of new psychiatric disorder diagnoses (Bryant et al. 2010). However, less is known about the longer-term psychological burden after injury.

Internationally, experiences of psychological distress have been found to persist for at least two years after being hospitalised for injury (Saunders et al. 2012; Mayou and Bryant 2002; Pelissier et al. 2020; Sigurdardottir et al. 2013; Soberg et al. 2012) and often after reports of physical health improvements (Kruithof et al. 2020). For instance, in the Netherlands, a prospective longitudinal cohort study interviewed 1105 trauma patients hospitalised for a range of injuries, at one-week, one-, three-, six-, 12 and 24 months post-injury (Kruithof et al. 2020). The prevalence of depression (12%) and anxiety (10%) declined only slightly from one week to 24 months post-injury (7% and 8%, respectively). Additionally, post-traumatic stress remained stable and was significantly higher at 24-months (11%) than in the general Dutch population (2.6–3.3%).

In a longitudinal study in the USA, 801 adults with long-term spinal cord injury (SCI) averaging 15 years post-SCI, were assessed for clinically relevant distress at two timepoints over a five-year interval (Saunders et al. 2012). Although there was a decline over time, 22% had probable major depression (PMD) between 2002 and 2004, and over half of those still met the criteria for PMD five years later. In France, a prospective cohort study of 1168 adults with primarily mild to moderate injuries followed 691 adults (59%) to five years post-injury and found 24% were experiencing psychological distress (Pelissier et al. 2020).

Despite evidence that levels of psychological distress remain high several years after injury, studies have often relied on long and variable recall of pre-injury distress (Saunders et al. 2012), or were restricted by small numbers of injured people and high attrition at final follow-up (Pelissier et al. 2020). Few studies assessed distress at more than one timepoint (Saunders et al. 2012; Sigurdardottir et al. 2013; Soberg et al. 2012; Hoffman et al. 2011), and of those, only small numbers of patients were assessed at all timepoints. Finally, research has been limited to specific sub-groups of participants such as those hospitalised for injuries, certain injury types (e.g. SCIs, traumatic brain injuries) [2, 12, 15,] and causes (e.g. motor vehicle crashes) (Pelissier et al. 2020). Findings from these studies may not be generalisable to groups with other causes of injury or treated in non-hospital settings (Polinder et al. 2012). For example, injuries that have not resulted in hospitalisation are associated with reduced physical health, poorer quality of life (Derrett et al. 2013; Langley et al. 2013) and account for more than two-thirds of years lived with disability after injury (Lyons et al. 2011). It is important to understand the extent and duration of psychological distress following a range of injury types, causes and severities.

In our earlier Prospective Outcomes of Injury Study (POIS), 2856 New Zealanders with diverse injuries were followed over time to investigate the effects of multiple characteristics on a range of outcomes after a sentinel injury event (SIE) (Derrett et al. 2011). Participants were interviewed via telephone at 3, 12 and 24 months post-SIE. Previously, we explored the prevalence of psychological distress among POIS participants at 3, 12 and 24 months after their SIEs and identified factors that influenced psychological distress up to 24 months post-SIE (Richardson et al. 2021). The prevalence of psychological distress was 25%, 15% and 16% at those respective timepoints (Richardson et al. 2021). Several pre-injury, injury-related and early post-injury factors predicted psychological distress at 12 months and continued to predict distress at 24 months post-SIE. It is still unclear which factors are associated with longer-term distress. Therefore, this paper aims to: (1) describe the prevalence of psychological distress 12 years post-SIE and (2) investigate pre-injury, injury-related and early post-injury characteristics associated with long-term distress among injured New Zealand adults.

## Method

### Design and participants

Information on POIS recruitment and study design has been reported previously (Derrett et al. 2011, 2021). After sustaining an injury between 2007 and 2009, 2856 New Zealand citizens or residents (including 566 Māori) were randomly selected from the entitlement claims register of the Accident Compensation Corporation (ACC), New Zealand’s no-fault universal injury insurer and recruited to POIS. Participants sustained a wide range of injury types (e.g. fractures, sprains and strains) that occurred in a variety of settings (e.g. work, home, road) and from different causes (motor vehicle, assault, work-related) (Derrett et al. 2011). Māori (Indigenous peoples of New Zealand) comprise 17% of New Zealand’s population (Ministry of Health 2021a). POIS recruitment did not cease until 20% of the original cohort were injured Māori in order to understand long-term outcomes for this population who experience marked health inequities after injury, including for disability (Wyeth et al. 2019) and psychological distress (Richardson et al. 2021). Participants with injuries resulting from self-harm and sexual assault were ineligible for POIS. Using multiple contact details for participants and their significant others, interviewers made up to five attempts to contact participants via telephone, and if unsuccessful, participants were sent postal questionnaires (Derrett et al. 2011). The 2068 participants who completed a 24-month post-SIE interview and agreed to future follow-up and were not known as deceased, were invited to a follow-up interview, 12 years after their SIE (Derrett et al. 2021). Of the 2068 eligible participants, 1543 (75%) participants (including 240 Māori) took part in POIS-10 between 2020 and 2021. Most (*n* = 1430) completed a telephone interview; people who were unable to participate in a telephone interview due to time constraints or health reasons completed a hard copy questionnaire via free return mail (*n* = 113). Telephone interviews were approximately one hour in duration. Interviews were conducted before, during and after COVID-19 restrictions were introduced in NZ following the announcement of a nationwide Alert Level 4 COVID-19 lockdown from 25 March–26 April 2020. Ethical approval was obtained from the Health and Disability Ethics Committee New Zealand (MEC/07/07/093/AM07).

### Outcome measure

The Kessler Psychological Distress Scale (K6) was the outcome of interest. This measure comprises six items and is widely used to screen for mental illness in the general population (Kessler et al. 2002, 2010). For each item, respondents are asked to indicate the extent that they experience distress over the past 30 days, using a five-point scale with response options ranging from 0 (i.e. none of the time) to 4 (All of the time). Items are summed to calculate a total score between 0 and 24 (Richardson et al. 2021; Wang et al. 2007). Total scores were not calculated for participants with at least one K6 item missing or a “don’t know” response.

K6 total scores were categorised into three levels distress severity: 0–7 (probable absence of mental illness); 8–12 (probable mild-moderate mental illness); and ≥ 13 (probable serious mental illness). As previously (Richardson et al. 2021), we treated scores of ≥ 8 as the cut-off for clinically relevant distress likely to warrant mental health intervention (Prochaska et al. 2012).

### Explanatory variables

As in our previous POIS analyses, potential explanatory (predictor) variables were investigated and presented according to four categories: socio-demographic and pre-injury, injury-related, health-service and early post-injury characteristics (Richardson et al. 2021).

#### Socio-demographic and pre-injury characteristics

Pre-injury socio-demographic characteristics were collected using New Zealand Census questions about age at time of injury, sex, ethnicity, living arrangements and highest educational qualification (Statistics New Zealand 2006). Ethnicity is presented using the prioritisation method and categories (i.e. Māori, Pacific, Asian, Other and NZ European) (Ministry of Health 2017). Living arrangements were categorised as ‘living alone or with non-family’ or ‘living with family, including with a partner/spouse’; education as ‘less than secondary school’ or ‘secondary school or higher’. Adequacy of household income to meet participants’ everyday needs was categorised as ‘adequate’ (‘enough’ or ‘more than enough’) or ‘inadequate’ (if people reported ‘not enough’ or ‘just enough’) (Ministry of Social Development 2000). Paid work before injury, was categorised as ‘Yes’ (‘full-time’ or ‘part-time’ work) and ‘No’ if not in paid work.

Participants reported whether they had been told by a doctor before their injury that they had one or more of a list of 21 long-term health conditions (4 mental and 17 physical) that lasted, or were expected to last, for 6-months or more (Ministry of Health 2006) and to report their general health using a 5-point scale (Ware et al. 2000). Pre-injury alcohol use was assessed using the AUDIT-C and categorised as ‘hazardous’ (AUDIT-C ≥ 3 for females; ≥ 4 for males) or ‘non-hazardous’ drinking (Bush et al. 1998).

#### Injury-related characteristics

Participants’ injuries, leading to POIS recruitment (2007–2009), were categorised according to severity using the New Injury Severity Score (NISS) based on each participant’s injury diagnosis data provided by ACC; categorised as NISS 1–3 (least severe), 4–6 (moderately severe), or > 6 (most severe) (Lavoie et al. 2004; Wilson et al. 2014). Participants also reported whether, at the time of their injury, their injury was intentional (i.e. assault) or unintentional, and whether they perceived the injury to be a threat to their life or not (‘yes’ or ‘no’).

#### Health service-related characteristics

Three months post-SIE, participants rated experiences of healthcare services and contact with ACC on a 5-point scale (‘very good’ to ‘very bad’). Participants also reported trouble getting to or contacting health services for their injury.

#### Early post-injury characteristics (3 months)

At the 3-month interview, early post-injury K6 was collected and participants were also asked about their expectations for injury recovery (‘completely recovered’, ‘get better soon/slowly’, ‘don’t know’/ ‘don’t know future course’ or ‘never get better’). Satisfaction with social relationships was categorised as ‘satisfied’ (‘completely satisfied’ or ‘mostly satisfied’) and ‘not satisfied’ (if people reported ‘don’t know’, ‘neither satisfied nor dissatisfied’, ‘mostly dissatisfied’ or ‘completely dissatisfied’).

### Statistical analyses

Data were analysed using Stata SE 17 (Statistical and Software 2021). Cross-tabulations separately describe characteristics of participants who reported clinically relevant distress (K6 ≥ 8) at 12 years post-SIE from those who did not. The univariable analysis of distress at 12 years was conducted for each pre-injury, injury-related, health service-related and early post-injury characteristic in univariable modified Poisson regressions, using robust standard errors (Zou 2004). A modified multivariable Poisson regression model was then developed to identify which characteristics predicted an increased (or reduced) risk of distress at 12 years post-SIE, accounting for all other variables. A stepwise backward selection method with a *p*-value threshold of ≤ 0.10 was used to identify the least significant variable to remove from each step of the multivariable model, until the final model contained only characteristics that were significantly associated with an increased (or reduced) risk of distress. Sex, age at time of injury and ethnicity were held constant at each step in the model building process. Only participants who had complete data for relevant explanatory measures were included in the final model.

## Results

Of 1543 participants recruited to POIS-10, 1526 provided complete K6 outcome data at 12 years post-SIE (99%). Table 1 provides the number of participants who reported mild-moderate distress or serious distress at 12 years post-SIE. The prevalence of clinically relevant distress at 12 years post-SIE (12%) appears to have reduced slightly from the prevalence 24 months post-SIE (16%).

**Table 1:** Participants reporting clinically relevant distress at 3, 12, 24 months and 12 years post-SIE

	3 months *n* = 2821 *n* (%)	12 months *n* = 2239 *n* (%)	24 months *n* = 2217 *n* (%)	12 years *n* = 1526 *n* (%)
*Severity of distress (K6)*				
Absence (≤ 7)	2122 (75)	1906 (85)	1864 (84)	1349 (88)
Mild-moderate (8–12)	464 (17)	249 (11)	259 (12)	126 (8)
Serious (≥ 13)	235 (8)	84 (4)	94 (4)	51 (4)
*Clinically relevant distress (*≥ *8)*				
No	2122 (75)	1906 (85)	1864 (84)	1349 (88)
Yes	699 (25)	333 (15)	353 (16)	177 (12)

Table 2 presents pre-injury, injury-related, health service-related and post-injury characteristics of participants according to whether or not they reported clinically relevant distress. Descriptively, differences included a greater proportion of Māori, Asian or Other ethnicities reported distress (19% each) compared to those of European ethnicity (8%), and a greater proportion of those reporting an inadequate household income pre-injury (18%) reported distress compared to those with an adequate income (9%). Also, a greater proportion of people who experienced early post-injury distress reported distress 12 years post-SIE (26%) compared to people who did not experience early post-injury distress (7%).

**Table 2:** Socio-demographic, injury-related and early post-injury characteristics of distressed and non-distressed participants at 12 years post-SIE

	Not distressed (*n* = 1349) *n* (%)	Distressed (*n* = 177) *n* (%)	*P* value
*Pre-injury characteristics*			
Sex			
Male	761 (89)	95 (11)	
Female	588 (88)	82 (12)	
Age in years (at time of injury)			
18–24	130 (88)	18 (12)	< 0.01
25–34	240 (86)	40 (14)	
35–44	307 (86)	52 (14)	
45–54	394 (89)	49 (11)	
55–65	278 (94)	18 (6)	
Ethnicity			
Māori	195 (81)	43 (19)	< 0.001
Pacific	36 (86)	6 (14)	
Asian	70 (81)	16 (19)	
Other	166 (81)	38 (19)	
NZ European	882 (92)	74 (8)	
Education			
Less than secondary school	283 (83)	57 (17)	< 0.01
Secondary school or higher	1047 (90)	117 (10)	
Living arrangements			
Alone/with non-family	214 (86)	36 (14)	0.14
With family	1131 (89)	141 (11)	
Income adequacy			
Adequate	956 (91)	91 (9)	
Inadequate	384 (82)	84 (18)	
Working for pay			
No	90 (84)	17 (16)	0.15
Yes	1258 (89)	160 (11)	
Mental health conditions			
0	1182 (90)	128 (10)	< 0.001
1	81 (70)	34 (30)	
≥ 2	39 (76)	12 (24)	
Physical health conditions			
0	729 (90)	78 (10)	< 0.01
1	375 (87)	54 (13)	
≥ 2	198 (83)	42 (18)	
General health			
Excellent/very good	972 (91)	96 (9)	< 0.001
Good	325 (84)	62 (16)	
Fair/poor	50 (72)	19 (28)	
Alcohol use			
Non-hazardous	449 (87)	70 (13)	0.1
Hazardous	889 (89)	106 (11)	
*Injury-related characteristics*			
Injury severity (NISS)			
1–3 (least severe)	514 (86)	82 (14)	0.1
4–6 (severe)	645 (90)	70 (10)	
≥ 7 (most severe)	149 (89)	18 (11)	
Cause of injury			
Unintentional	1303 (89)	163 (11)	< 0.01
Intentional (assault)	39 (74)	14 (26)	
Perceived threat to life			
No	1213 (89)	145 (11)	< 0.001
Yes	115 (79)	30 (21)	
*Health service characteristics*			
Health service experience			
Very good/good/moderate	1305 (89)	165 (11)	0.04
Bad/very bad	38 (79)	10 (21)	
ACC experience			
Very good/good/moderate	1213 (89)	154 (11)	0.12
Bad/very bad	75 (83)	15 (17)	
Access to health services			
No trouble	1185 (88)	160 (12)	0.25
Trouble/mixed	154 (91)	15 (9)	
*Early post-injury characteristics*			
Distress 3 months post-SIE (K6 ≥ 8)			
Not Distressed	1087 (93)	87 (7)	< 0.001
Distressed	259 (74)	89 (26)	
Expectations for recovery			
Already recovered	286 (93)	20 (7)	< 0.001
Expect to recover soon/slowly	825 (89)	103 (11)	
Don’t know future course	186 (80)	46 (20)	
Expect to never recover	46 (88)	6 (12)	
Satisfaction with social relationships			
Satisfied	1180 (91)	121 (9)	< 0.001
Neutral/dissatisfied	165 (75)	55 (25)	

Column totals for each variable vary as missing values have been omitted. Percentages may not always add to 100 due to rounding. With the exception of Expectations of Recovery and Satisfaction with Social Relationships, “don’t know” and “not applicable” responses were treated as missing. Missing response categories were omitted from calculations of Pearson’s*c*^2^ and *p*-values are presented above

*SIE* sentinel injury event

Table 3 presents univariable analyses estimating the relative risk of distress at 12 years post-SIE of different pre-injury, injury-related and early post-injury characteristics. Several pre-injury, injury-related, health-related and early post-injury characteristics were separately associated with increased risk of clinically relevant distress 12 years post-SIE. Specifically, injured Māori and Asian people had over twice the risk of psychological distress compared to Europeans (RR = 2.33; 95% CI 1.65, 3.31 and RR = 2.40; 95% CI 1.47, 3.93, respectively). Inadequate pre-injury household income also doubled the risk of distress at 12 years compared to those with an adequate income (RR = 2.07; 95% CI 1.57, 2.72). Participants with one, or more, pre-injury mental health conditions were more than twice as likely to report distress (RR = 3.03; 95% CI 2.18, 4.19 and RR = 2.41; 95% CI 1.43, 4.06, respectively) and having one, or more, pre-injury physical health conditions also increased the risk (RR = 1.30; 95% CI = 0.94, 1.81 and RR = 1.81; 95% CI 1.28, 2.56). Compared to those who perceived their pre-injury health to be ‘excellent’ or ‘very good’, people reporting ‘good’ pre-injury health were almost twice as likely to experience distress at 12 years (RR = 1.78; 95% CI 1.32, 2.40), and reporting ‘fair’ or ‘poor’ health tripled the risk of distress (RR = 3.06; 95% CI 2.00, 4.70). Sustaining an intentional injury (i.e. due to assault) more than doubled the risk of distress at 12 years compared to those who sustained an unintentional injury (RR = 2.38; 95% CI 1.48, 3.81). Similarly, people who perceived their injuries to be life threatening were also almost twice as likely to report distress (RR = 1.94; 95% CI 1.36, 2.76). Having a ‘bad’ or ‘very bad’ experience with healthcare services increased the risk of distress at 12 years compared to having a ‘moderate’, ‘good’ or ‘very good’ experience (RR = 1.86; 95% CI 1.05, 3.28). People who experienced early post-injury distress were more than twice as likely to experience distress at 12 years than those who did not (RR = 3.45; 95% CI 2.63, 4.52), as were participants who were not satisfied with social relationships compared to those who were satisfied (RR = 2.69; 95% CI 2.02, 3.57). Having (at a minimum) a secondary school education qualification reduced the risk of distress at 12 years by approximately 40% compared to those without a secondary school education (RR = 0.6; 95% CI 0.45, 0.80). People who were 45–65 years were more than a third less likely to report distress at 12 years compared to participants aged 18–44 years (RR = 0.65; 95% CI 0.49, 0.86) were associated with reduced risk of distress.

**Table 3:** Univariable associations between socio-demographic, injury-related and post-injury characteristics with distress at 12 years post-SIE (*n* = 1526)

	Distress at 12 years		
	RR	95% CI for RR	*P* value
*Pre-injury characteristics*			
Sex			
Male	Ref		
Female	1.10	0.84, 1.46	0.49
Age in years (at time of injury)			
18–44	Ref		
45–65	0.65	0.49, 0.86	< 0.01
Ethnicity			
NZ European	Ref		
Māori	2.33	1.65, 3.31	< 0.001
Pacific	1.85	0.85, 4.00	
Asian	2.40	1.47, 3.93	
Other	2.40	1.68, 3.45	
Education			
Less than secondary school	Ref		
Secondary school or higher	0.60	0.45, 0.80	< 0.01
Living arrangements			
Alone/with non-family	Ref		
With family	0.77	0.55, 1.08	0.13
Income adequacy			
Adequate	Ref		
Inadequate	2.07	1.57, 2.72	< 0.001
Working for pay			
No	Ref		
Yes	0.71	0.45, 1.12	0.15
Mental health conditions			
0	Ref		
1	3.03	2.18, 4.19	
≥ 2	2.41	1.43, 4.06	< 0.001
Physical health conditions			
0	Ref		
1	1.30	0.94, 1.81	
≥ 2	1.81	1.28, 2.56	< 0.01
General health			
Excellent/very good	Ref		
Good	1.78	1.32, 2.40	
Fair/poor	3.06	2.00, 4.70	< 0.001
Alcohol use			
Non-hazardous	Ref		
Hazardous	0.79	0.60, 1.05	0.1
*Injury-related characteristics*			
Injury severity (NISS)			
1–3 (least severe)	Ref		
4–6 (severe)	0.71	0.53, 0.96	
≥ 7 (most severe)	0.78	0.48, 1.27	0.1
Cause of injury			
Unintentional	Ref		
Intentional (assault)	2.38	1.48, 3.81	< 0.001
Perceived threat to life			
No	Ref		
Yes	1.94	1.36, 2.76	< 0.001
*Health service characteristics*			
Health service experience			
Very good/good/moderate	Ref		
Bad/very bad	1.86	1.05, 3.28	0.03
ACC experience			
Very good/good/moderate	Ref		
Bad/very bad	1.48	0.91, 2.40	0.11
Access to health services			
No trouble	Ref		
Trouble/mixed	0.75	0.45, 1.24	0.26
*Early post-injury characteristics*			
Distress 3 months post-SIE (K6 ≥ 8)			
Not distressed	Ref		
Distressed	3.45	2.63, 4.52	< 0.001
Expectations for recovery			
Already recovered	Ref		
Expect to recover soon/slowly	1.70	1.07, 2.69	
Unsure	3.03	1.85, 4.98	
Expect to never recover	1.77	0.74, 4.19	< 0.001
Satisfaction with social relationships			
Satisfied	Ref		
Neutral/dissatisfied	2.69	2.02, 3.57	< 0.001

For predictors with more than two categories, *p*-values are reported for a joint test. There were no missing responses for sex, age and ethnicity. Most variables were missing between 1–23 participant responses, with the exception of mental health conditions, physical health conditions, NISS and ACC experience that were missing 48–69 responses

*SIE* sentinel injury event

The relative risks associated with increased (or reduced) distress at 12 years are presented in the final multivariable model (Table 4). Variables retained in the final model were age at time of injury, ethnicity, pre-injury income adequacy and experience of mental health conditions, rating of experience with ACC, trouble accessing health services and distress (K6 ≥ 8) and satisfaction with social relationships at three-months post-injury. Participants who were older (aged 45–65 years) were 28% less likely to experience distress at 12 years than participants who were younger (18–44 years) (RR = 0.72; 95% CI 0.55, 0.96). Māori participants had approximately twice the risk of distress compared to NZ Europeans (RR = 2.18; 95% CI 1.54, 3.10). Pacific participants (RR = 2.12; 95% CI 1.04, 4.34), Asian participants (RR = 2.62; 95% CI 1.63, 4.20) and people with Other ethnicities (RR = 2.43; 95% CI 1.66, 3.56) were also at increased risk of distress compared to NZ Europeans. Those who reported an inadequate household income pre-injury were at an increased risk of distress compared to those with adequate income (RR = 1.62; 95% CI 1.22, 2.14). Participants with one pre-injury diagnosed mental health condition were at increased risk of distress compared to those who had no diagnosed mental health conditions before their injury (RR = 2.28, 95% CI 1.60, 3.25). Those who reported clinically relevant distress (K6 ≥ 8) at the three-month interview were at increased risk of distress at 12 years post-SIE (RR = 2.35; 95% CI 1.70, 3.25). Furthermore, participants who were not satisfied with social relationships at three months post-SIE were also at increased risk of distress compared to those who were satisfied (RR = 1.47; 95% CI 1.06, 2.03).

**Table 4:** Multivariable model estimating relative risk of distress 12 years post-SIE for pre-injury, injury-related and post-injury characteristics (*n* = 1391)

	Distress at 12 years		
	RR	95% CI for RR	*P* value
*Pre-injury characteristics*			
Sex			
Male	Ref		
Female	1.05	0.80, 1.38	0.72
Age in years (at time of injury)			
18–44	Ref		
45–65	0.72	0.55, 0.96	0.03
Ethnicity			
NZ European	Ref		
Māori	2.18	1.54, 3.10	< 0.001
Pacific	2.12	1.04, 4.34	
Asian	2.62	1.63, 4.20	
Other	2.43	1.66, 3.56	
Income adequacy			
Adequate	Ref		
Inadequate	1.62	1.22, 2.14	< 0.01
Mental health conditions			
0	Ref		
1	2.28	1.60, 3.25	< 0.001
≥ 2	1.57	0.88, 2.80	
*Health service characteristics*			
ACC experience			
Very good/good/moderate	Ref		
Bad/very bad	1.38	0.89, 2.14	0.15
Access to health services			
No trouble	Ref		
Trouble/mixed	0.61	0.36, 1.05	0.07
*Early post-injury characteristics*			
Distress 3 months post-SIE (K6 ≥ 8)			
Not distressed	Ref		
Distressed	2.35	1.70, 3.25	< 0.001
Satisfaction with social relationships			
Satisfied	Ref		
Not satisfied	1.47	1.06, 2.03	0.02

For predictors with more than two categories, *p*-values are reported for a joint test. Sex, age and ethnicity were held constant. Prioritised ethnicity was used (Ministry of Health 2017)

*SIE* sentinel injury event

## Discussion

Clinically relevant distress was reported by 12% of POIS-10 participants 12 years post-SIE. Several socio-demographic factors (age, ethnicity, income adequacy), pre-injury mental health conditions and early post-injury factors (i.e. distress and satisfaction with social relationships) were associated with distress at 12 years. Injury severity or cause did not appear to be related to long-term distress in multivariable analyses. To our knowledge, this is the first prospective cohort study exploring the prevalence of long-term psychological distress (beyond two years) among those who have sustained a range of injuries, including those typically considered a “low threat to life” (Derrett et al. 2021).

In our earlier analyses, the prevalence of psychological distress was 25% at 3 months post-SIE, 15% at 12 months post-SIE and 16% at 24 months post-SIE (Richardson et al. 2021). When restricting the present sample to only those with data at each of these timepoints, the prevalence of distress was very similar to that reported previously. At 12 years post-SIE, the proportion distressed (12%) is higher than in the general New Zealand population (8%) (Ministry of Health 2021b). This is consistent with findings from studies in other countries that the prevalence of psychological distress declines over time but remains higher among injured adults than in the general population several years after injury (Saunders et al. 2012; Pelissier et al. 2020; Hoffman et al. 2011).

Nevertheless, the prevalence of psychological distress in POIS-10 was lower than in other studies with different injury populations. For example, the prospective cohort study of 691 adults who sustained traffic injuries in France found that 24% experienced psychological distress five years post-injury (Pelissier et al. 2020), while longitudinal studies of trauma patients (i.e. SCI and TBI) in USA and Norway found that 18% were distressed at five years (Sigurdardottir et al. 2013; Hoffman et al. 2011). These differences in the long-term prevalence of distress between studies could be accounted for by different measurement scales. For instance, the French prospective cohort study used the 12-item General Health Questionnaire, which has been found to have lower reliability and validity than the K6 (Cornelius et al. 2013). Nevertheless, the Norwegian longitudinal study measured distress using the Hospital Anxiety and Depression Scale (Sigurdardottir et al. 2013), which can result in more conservative estimates of distress and a higher rate of false negatives (Cosco et al. 2012), and yet the prevalence of distress was still higher compared to the present study. In contrast to these studies, POIS-10 has followed participants to a longer time post-injury, and our participants’ injuries were treated in a range of settings (only 25% were originally hospitalised for injury treatment) (Derrett et al. 2013).

This study identified several socio-demographic and pre-injury characteristics associated with an increased risk of clinically relevant distress at 12 years, including for Māori, Pacific or Asian ethnic groups and having inadequate income. Previous POIS research found that Māori experience more adverse mental health (in addition to physical health) outcomes after injury compared to non-Māori, including an increase in clinically relevant symptoms (Maclennan et al. 2013) and a higher prevalence of psychological distress at 12 and 24 months post-injury (Richardson et al. 2021; Maclennan et al. 2014). Our study indicates that Māori and Asian participants are at higher risk of long-term distress, potentially even after physical recovery from injury. At 12 years post-SIE, 19% of both Māori and Asian participants reported clinically relevant distress compared to 8% of NZ Europeans. Māori mental health inequities may be attributed to the practical barriers faced in accessing health services (e.g. cost, distance, lack of culturally safe or appropriate services), lack of clarity about health information provided and experiences of fragmented and discriminatory care (Espiner et al. 2021). These factors are also exacerbated by intergenerational impacts of colonisation and systemic racism (Graham and Masters-Awatere 2020).

Having one pre-injury mental health conditions was also associated with increased risk of distress among injured POIS-10 participants at 12 and 24 months (Richardson et al. 2021) and at 12 years post-SIE. However, being older at the time of the injury event, was associated with reduced risk of distress 12 years post-SIE; although not at 12 or 24 months post-SIE (Richardson et al. 2021). Conversely, pre-injury physical health conditions, fair/poor general health and trouble getting to or contacting health services were associated with an increased risk of distress 24-months post-SIE but were not significant predictors longer-term.

Regarding early post-injury characteristics, not reporting satisfaction with social relationships at 3 months post-SIE was associated with increased risk of distress at 12 years post-SIE. Having strong social support can mitigate negative impacts of injury long-term. This is particularly common in Māori communities where whānau often carry some of the burden and provide social, emotional and spiritual support (Dew et al. 2015). Also, participants who experienced early post-injury distress had more than double the risk of experiencing distress at 12 years. This is consistent with international findings that distress may persist overtime, or that even if an individual’s mental health improves, they may still experience clinically relevant distress in future (Sigurdardottir et al. 2013).

There were no associations observed between injury severity (measured by NISS) and injury cause with distress at 12 years post-SIE, which was somewhat expected given that these factors were only associated with distress at 12 months post-SIE but not at 24 months in our cohort (Richardson et al. 2021). In contrast, internationally, injury severity has been found to be associated with distress at 12 months (Kendrick et al. 2017) and four years post-injury (Tran et al. 2016) and sustained over a five-year period, once behavioural factors were controlled for (e.g. sleep and exercise) (Saunders et al. 2012). There are still inconsistencies in the literature about the relative impact of anatomical injury severity on mental health compared to an individual’s perception of their injury (Pelissier et al. 2020). It is plausible that because the majority of POIS-10 participants’ injuries (89%) were perceived as a low threat to life and were unintentional (96%), injury-related factors had less impact on long-term distress.

The present study findings may not be generalisable to injured adults who sustained a particular injury type (e.g. TBIs, SCIs) in a specific event and setting (e.g. traffic versus work-related). For instance, an Australian prospective cohort study found that adults with TBIs reported greater psychological distress and psychosocial difficulties up to 10 years post-injury compared to adults with traumatic orthopaedic injuries (Dahm and Ponsford 2015). Also, because our cohort was aged between 18 and 65 years at the time of their injury, with the majority (92%) in paid employment, our findings may be less generalisable to older or younger populations.

Furthermore, POIS-10 achieved a 75% response rate; however, 525 potential POIS-10 participants did not complete an interview, and of these, 360 people could not be reached via telephone for an interview. In some instances, non-participation may be due to poor physical or mental health, or slow injury recovery, and thus, the prevalence of distress may be lower among POIS-10 participants than eligible non-participants. Also, with the exclusion of people with injuries sustained by self-harm and sexual assault for our original POIS recruitment in 2007–2009, the prevalence of psychological distress at 12 years is likely underestimated. Finally, this study may not have accurately captured fluctuations in distress overtime. Given the findings of USA and Norwegian longitudinal studies (Sigurdardottir et al. 2013; Soberg et al. 2012; Hoffman et al. 2011), it is plausible that even when some POIS-10 participants recover from distress, they may still experience re-occurring symptoms at a later timepoint. However, it is likely that only smaller proportions of participants meet the criteria for clinically relevant distress at all timepoints.

### Clinical implications

Other studies have found post-injury experiences of psychological distress have reduced physical functioning and quality of life (Kendrick et al. 2017) and increased the risk of developing serious psychiatric disorders (Bryant et al. 2010). Given that distress at 3 months post-SIE is a strong predictor of distress at 12 years, it is important that injured adults are screened for distress by their health provider as soon as possible after an injury and that socio-economic and early post-injury risk factors for distress are identified. US studies have reported low rates of mental health treatment for patients who met the clinical criteria for distress after a SCI (29%) or a TBI (22–31%) (Fann et al. 2011; Huebner et al. 2018). In our study, participants were not asked about their use of mental health services or prescribed medication for clinically relevant distress at the time of, or after, their injury. If distress screening could be provided through injury rehabilitation services (Perkes et al. 2014), injured individuals are more likely to be referred to mental health services that could aid their recovery and help reduce the likelihood of experiencing long-term distress.

## Conclusion

Internationally, psychological distress after injury is more common than among the general population, even several years after injury (Saunders et al. 2012; Pelissier et al. 2020; Hoffman et al. 2011). POIS-10 study findings indicate that the prevalence of distress after injury declines overtime but remains higher than in the general New Zealand population out to 12 years after injury. The study also identifies socio-demographic factors (i.e. age, ethnicity, pre-injury income adequacy), pre-injury mental health and early post-injury distress and satisfaction with social relationships as predictors of distress at 12 years, thus raising the importance of ongoing social and financial support for people at risk of distress, even prior to their injury. Our findings also highlight the need for early detection of, and interventions for, distress or distress symptoms and integration of healthcare services to prevent further development of more severe psychiatric disorders (Bryant et al. 2010).

## Data Availability

The datasets generated and/or analysed for this paper are not publicly available due to ethical constraints and potentially sensitive and identifiable data. If anyone is interested in pursuing collaborative research or discussing data sharing opportunities, please contact the corresponding author.
